# Behavior of serum thyroglobulin in relation to thyroid function under low-thyrotropin conditions in general practice

**DOI:** 10.3389/fendo.2026.1756863

**Published:** 2026-05-15

**Authors:** Yosuke Sazumi, Yoshiaki Soejima, Yuki Otsuka, Yasuhiro Nakano, Koichiro Yamamoto, Atsuhito Suyama, Ryosuke Takase, Kohei Oguni, Miho Yasuda, Masanori Furukawa, Fumio Otsuka

**Affiliations:** 1 Department of General Medicine, Okayama University Graduate School of Medicine, Dentistry and Pharmaceutical Sciences, Okayama, Japan; 2 Department of Laboratory Medicine, Okayama University Hospital, Okayama, Japan

**Keywords:** FT3/FT4 ratio, hyperthyroidism, iodothyronine deiodinase, thyroglobulin, TSH- receptor antibody

## Abstract

**Introduction:**

Serum thyroglobulin (Tg) and thyroid hormones are essential biomarkers in the diagnosis and management of thyroid diseases. Although Tg is routinely measured as a marker for thyroid neoplasia and an adjunct in the diagnosis of thyrotoxicosis due to destructive thyroiditis, its clinical significance under conditions of abnormal thyroid function remains unclear.

**Methods:**

We investigated the association between serum Tg levels and thyroid function in 292 patients who underwent simultaneous evaluation of serum Tg, free thyroxine (FT4), and free triiodothyronine (FT3) levels in routine clinical practice. Based on thyroid-stimulating hormone (TSH) levels, participants were classified into three groups: high-TSH (> 4.23 μIU/mL; n = 38), normal-TSH (0.61–4.23 μIU/mL; n = 191), and low-TSH (< 0.61 μIU/mL; n = 63).

**Results:**

The low-TSH group exhibited significantly higher serum Tg levels (118.66 ± 32.13 ng/mL) than the normal-TSH (70.20 ± 23.48 ng/mL) and high-TSH (34.70 ± 13.62 ng/mL) groups. Among patients positive for TSH-receptor antibodies (TRAb) or thyroid-stimulating antibodies (TSAb), elevated Tg levels were observed exclusively in the low-TSH group. Correlation analyses revealed that Tg levels were positively correlated with the FT3/FT4 ratio in the low-TSH group (*rho* = 0.62, *p* < 0.01), and this association was more evident in patients positive for both TRAb and TSAb (*rho* = 0.71, *p* < 0.01). These associations remained significant after multivariable adjustment and were supported by correlations between Tg and SPINA-GD, a model-based index of thyroid function reflecting deiodinase activity, with similar findings observed in patients with autoimmune hyperthyroidism.

**Conclusion:**

These findings suggest that serum Tg levels may reflect biochemical features consistent with T3 predominance in autoimmune hyperthyroidism and have potential clinical utility in characterizing disease pathophysiology.

## 1 Introduction

Thyroglobulin (Tg) is a 660-kDa dimeric glycoprotein synthesized exclusively by the thyroid follicular cells and stored in the follicular lumen (1). Physiologically, Tg serves as both an iodine storage protein and precursor for thyroid hormone synthesis. Iodide is actively transported into the thyroid via the sodium/iodide symporter and subsequently oxidized by thyroid peroxidase (TPO) before incorporation into Tg to form monoiodotyrosine (MIT) and diiodotyrosine (DIT). The coupling of MIT and DIT yields triiodothyronine (T3) and thyroxine (T4), which are released into the circulation upon stimulation by thyroid-stimulating hormone (TSH) (2). Although Tg is normally confined within the thyroid follicles, elevated serum Tg concentrations can occur under certain physiological or pathological conditions.

Serum Tg and thyroid hormone concentrations are fundamental biochemical parameters for diagnosing and managing a wide range of thyroid disorders. Routine measurement of serum Tg has become an integral component of clinical practice, serving as a sensitive biomarker for thyroid neoplasia and useful adjunct in differentiating thyrotoxicosis caused by destructive thyroiditis from other etiologies of thyroid hormone excess. In general practice, thyroid function tests, including Tg, are frequently performed for nonspecific symptoms such as fatigue, palpitations, weight changes, or goiter leading to the incidental discovery of latent thyroid dysfunction (3–6). Under conditions of normal thyroid function, circulating Tg levels are generally considered reliable indicators of thyroidal activity and structural integrity.

Nevertheless, in pathological states such as hyperthyroidism or hypothyroidism, interpretation of Tg levels becomes more complex. Fluctuations in serum Tg may not directly correspond to specific disease processes, making it challenging to determine whether these changes reflect tumor burden, degree of thyroid tissue destruction, or compensatory alterations in hormone synthesis. Furthermore, Tg has recently gained clinical relevance in the evaluation of immune checkpoint inhibitor–related thyroid dysfunction (1). Therefore, understanding the physiological and pathological determinants of Tg variability is crucial to ensure accurate clinical interpretation and optimize its diagnostic utility across diverse thyroid conditions.

Serum Tg levels reflect the presence and function of thyroid tissue, where Tg plays a central role in thyroid hormone synthesis and secretions (7). Previous studies have investigated associations between serum Tg and various thyroid conditions. Tg is widely used as a tumor marker for detecting recurrence or metastasis after surgery for differentiated thyroid carcinoma (8–10) and has been proposed as a biomarker of iodine deficiency in epidemiological studies (11–13). In rare cases, elevated Tg levels have led to the diagnosis of ectopic thyroid tissue, such as struma ovarii (14–16). Furthermore, a recent retrospective study (17) reported that serum Tg, but not TSH, was associated with an increased risk of thyroid cancer in patients undergoing thyroid nodule surgery, emphasizing the importance of considering TSH status when evaluating Tg as a biomarker.

In the present study, we classified patients according to thyroid function based on TSH levels and analyzed the relationship between Tg concentrations and thyroid hormone parameters, particularly the free triiodothyronine to free thyroxine conversion (FT3/FT4) ratio, in a general clinical setting.

## 2 Materials and methods

### 2.1 Study design and participants

We retrospectively reviewed the laboratory data of 373 patients who visited the Department of General Internal Medicine at Okayama University Hospital and underwent serum Tg measurement during hospitalization or outpatient care between April 1, 2021, and March 31, 2025. Of these, 81 patients were excluded due to the absence of simultaneous same-day measurements of serum TSH, FT3, FT4, and Tg. Ultimately, 292 patients were included in the analysis. Evaluation of thyroid function was conducted when thyroid dysfunction was clinically suspected, based on symptoms, medical history, or other laboratory findings. For patients who underwent multiple thyroid function assessments, only data from the first evaluation at the initial visit were included in the analysis. In addition, subgroup analyses were conducted in selected patient subsets, including those without levothyroxine treatment, patients in the Low-TSH group, antibody-positive patients, and those with available ultrasonographic data. These subgroup analyses were performed to further explore the relationships between serum Tg and thyroid-related parameters under specific clinical conditions.

### 2.2 Analysis of clinical parameters

The following laboratory data were obtained from patients’ medical records: TSH, FT4, FT3, FT3/FT4 ratio, Tg, TSH-receptor antibody (TRAb), thyroid-stimulating antibody (TSAb), anti-TPO antibody (TPOAb), and anti-Tg antibody (TgAb). TSAb levels were measured by BML, Inc. (Tokyo, Japan) using both bioassay and enzyme immunoassay methods between April 1, 2021, and March 31, 2023, and by bioassay alone between April 1, 2023, and March 31, 2025. All other laboratory tests were performed using automated analyzers operated by the central laboratory of Okayama University Hospital. TSH, FT3, FT4, and TRAb were measured using electrochemiluminescence immunoassay on the cobas 8000 analyzer (Roche Diagnostics. Switzerland) with Elecsys reagents. The assay used in this study (Roche Diagnostics) has a correction factor of 1.00; therefore, the measured values were used without adjustment. Tg, TPOAb, and TgAb were measured using chemiluminescent enzyme immunoassay on the LUMIPULSE L2400 system (Fujirebio Inc. Tokyo, Japan) with Lumipulse Presto reagents. Serum Tg was measured using an assay based on the Immunoassay for Total Antigen Including Complex via Pre-Treatment principle (18). Because reference ranges for TSAb varied depending on the assay used, we used a cutoff value <120%. For laboratory data reported as below or above the detection limits, the corresponding detection limit values were used for analysis. Thyroid volume was calculated using Brunn’s formula (width × length × depth × 0.479) in patients with available ultrasonographic measurements (19). Structure Parameter Inference Approach-derived thyroid’s secretory capacity (SPINA-GT) and SPINA-derived sum activity of peripheral deiodinases (SPINA-GD) were calculated using the SPINA Thyr model (RRID: SCR_014352; DOI: 10.5281/zenodo.3596049) (20). These parameters were used to assess thyroid secretory capacity and peripheral deiodinase activity, respectively, and were included in subsequent correlation analyses.

### 2.3 Statistical analyses

Participants were stratified into three groups according to their serum TSH levels based on the reference range used in our institutional laboratory: Low-TSH (<0.61 μIU/mL), Normal-TSH (0.61–4.23 μIU/mL; reference range), and High-TSH (>4.23 μIU/mL). The reference range for TSH reflects harmonized measurements in Japan, where standardization across different assay systems has been implemented since 2020 in accordance with initiatives by the International Federation of Clinical Chemistry and Laboratory Medicine (IFCC). Based on IFCC-aligned calibration (Phase IV), the reference interval for the Japanese adult population has been established as 0.61–4.23 μIU/mL. Scatter plots were generated to illustrate the relationships between serum Tg and other clinical parameters, including FT3, FT4 and FT3/FT4 ratio. The normality of all variables was assessed using the Shapiro–Wilk test, and as none followed a normal distribution, correlations were evaluated using Spearman’s rank correlation coefficient. The Kruskal–Wallis test was employed to compare group differences. If the result indicated a significant difference, the Steel-Dwass test was then used to compare all parameters against all other parameters. Statistical significance was defined as a *p* < 0.05.

Multivariable linear regression analyses were conducted with the FT3/FT4 ratio as the dependent variable and serum Tg as the primary independent variable. Both the FT3/FT4 ratio and Tg were log-transformed prior to analysis to improve the distribution of residuals. A crude model was first constructed, followed by Model I adjusted for age, sex, and FT4. Model II additionally included TRAb status, and Model III included TSAb status. Antibody status was incorporated into the regression models using dummy variables. Both TRAb and TSAb were categorized as positive, negative, or not measured. For each antibody, two dummy variables were created: one for positivity (coded as 1 for positive and 0 for negative or not measured) and another for missingness (coded as 1 for not measured and 0 for positive or negative), with the negative group as the reference. This approach allowed retention of cases with missing antibody data. Regression coefficients (*β*) and *p*-values were reported for each model. Statistical significance was defined as a *p* < 0.05.

All statistical analyses were performed using EZR (Jichi Medical University, Tochigi, Japan), a graphical user interface for R (The R Foundation for Statistical Computing, Vienna, Austria) (21). EZR is a modified version of R Commander, customized to include statistical functions commonly used in biostatistics.

### 2.4 Ethical approval and informed consent

All blood tests were performed in patients with adequate health insurance coverage. Study details were publicly disclosed on the hospital’s website, and patients were provided the opportunity to opt out. The study was performed in compliance with the principles of the Declaration of Helsinki and was approved by the Ethics Committee of Okayama University Hospital (approval no. 2508-022).

## 3 Results

### 3.1 Baseline characteristics of the study population

A total of 292 patients were included in the primary analysis based on baseline measurements. The study population consisted of 66 men (22.6%) and 226 women (77.4%), with a mean age of 56.29 ± 17.75 years. The age distribution was as follows: 55 patients (18.84%) were <40 years, 94 (32.19%) were 40–59 years, and 143 (48.97%) were ≥60 years. No pediatric patients (<15 years) were included in this study.

With regard to clinical background, 59 patients (20.21%) were diagnosed with Graves disease, 81 (27.74%) with Hashimoto thyroiditis, and 152 (52.05%) with other conditions. Three patients (1.03%) were pregnant at the time of evaluation (gestational weeks: 15, 18, and 30). Based on guideline-recommended trimester-specific considerations, their TSH values were reviewed, and none were reclassified into different TSH categories. Regarding treatment status, 58 patients (19.86%) were receiving levothyroxine, with a median dose of 75 μg/day [IQR: 37.50–109.38]. Antithyroid drugs were used in a minority of patients, including methimazole in 27 (9.25%) and propylthiouracil in 5 (1.71%). The detailed baseline characteristics are summarized in **Table 1**.

**Table 1:** Baseline characteristics of the study population.

Demographics	Number (%)
Sex, male (%)	66 (22.6)
Sex, female (%)	226 (77.4)
Age, mean ± SD	56.29 ± 17.75
Age categories, n (%)	
<40	55 (18.84)
40–59	94 (32.19)
≥60	143 (48.97)
Backgrounds of thyroid diseases	Number (%)
Pregnancy, n (%)	3 (1.03)
Final diagnosis	
* Graves disease*	59 (20.21)
* Hashimoto thyroiditis*	81 (27.74)
* Others*	152 (52.05)
Treatment for thyroid disorders	Number (%)
Levothyroxine use, n (%)	58 (19.86)
Dose (μg/day), median [IQR]	75 [37.50 – 109.38]
Antithyroid drug use	
Methimazole use	27 (9.25)
Propylthiouracil use	5 (1.71)

Data are presented as mean ± standard deviation (SD), median [interquartile range (IQR)], or number (%), as appropriate. Values in parentheses () indicate percentages unless otherwise specified. Values in square brackets [] represent the interquartile range (IQR). Age categories were defined as <40, 40–59, and ≥60 years. “Others” in the final diagnosis category includes adenomatous goiter, follicular thyroid carcinoma, drug-induced thyroid dysfunction, central hyperthyroidism, and central hypothyroidism. Graves disease includes patients in remission following antithyroid drug therapy. Hashimoto thyroiditis includes cases of painless thyroiditis occurring on a background of Hashimoto thyroiditis. IQR, interquartile range.

### 3.2 Distribution of serum thyroid hormone levels

**Figure 1:**
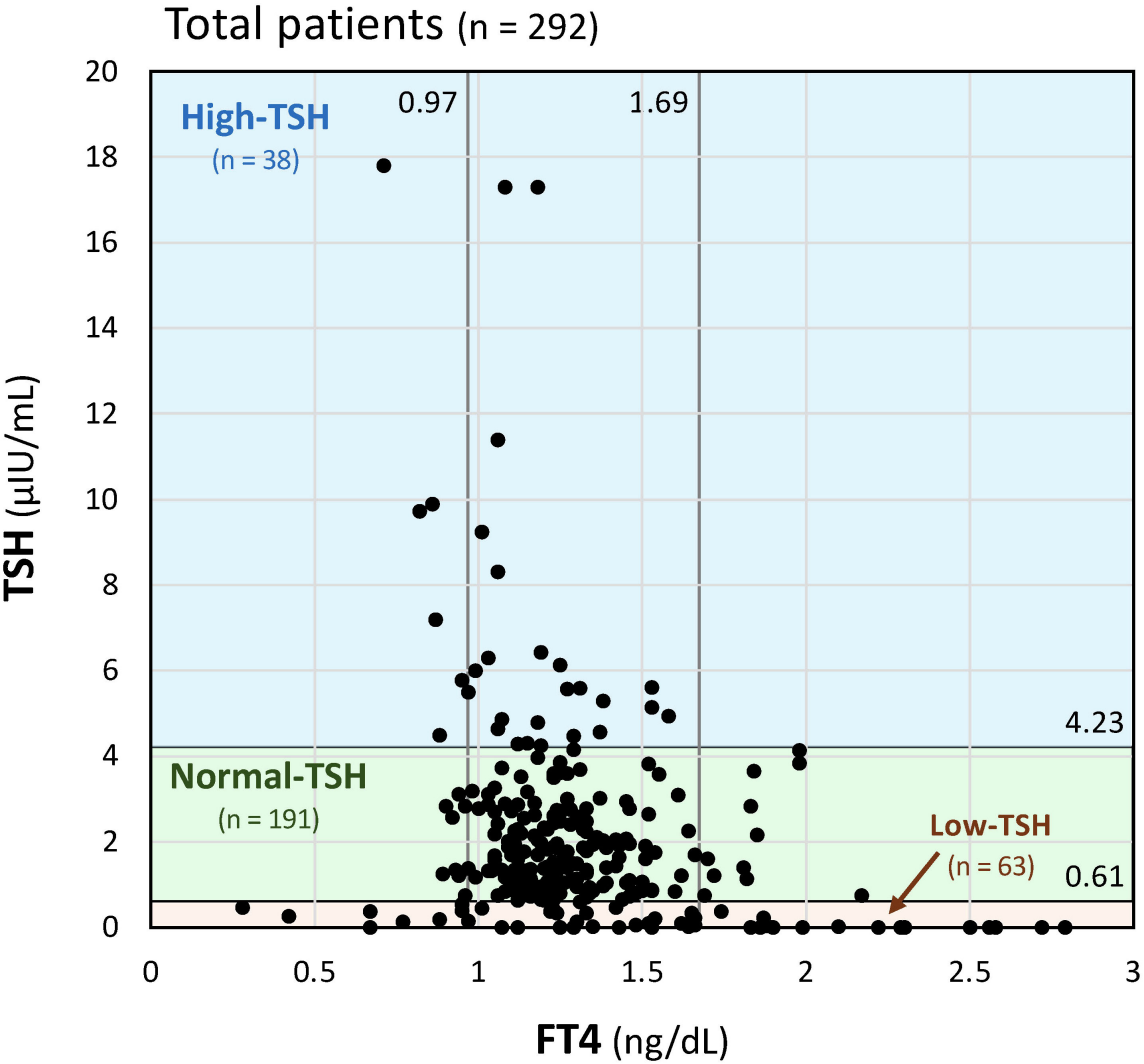
Distribution of serum thyroid-stimulating hormone (TSH) and free thyroxine (FT4) levels among the study population. Patients were categorized into three groups based on serum TSH concentrations: High-TSH (> 4.23 μIU/mL), Normal-TSH (0.61–4.23 μIU/mL), and Low-TSH (< 0.61 μIU/mL). Distribution of serum TSH and FT4 levels among the 292 patients included in the study. The High-, Normal-, and Low-TSH groups comprised 13% (n = 38), 65% (n = 191), and 22% (n = 63) of the total population, respectively.

**Figure 1** presents the distribution of serum TSH and FT4 levels in the study population. Among the 292 patients analyzed (**Figure 1**), 13% (n = 38), 65% (n = 191), and 22% (n = 63) were classified into the High-TSH, Normal-TSH, and Low-TSH groups, respectively.

As shown in **Table 2**, thyroid hormone levels differed significantly among the three TSH-defined groups. Median FT3 and FT4 concentrations in the Low-TSH group were 3.65 pg/mL and 1.66 ng/dL, respectively, compared with 2.84 pg/mL and 1.23 ng/dL, respectively, in the Normal-TSH group, and 2.57 pg/mL and 1.07 ng/dL, respectively, in the High-TSH group. Median TSH levels were 0.01 μIU/mL in the Low-TSH group, 1.65 μIU/mL in the Normal-TSH group, and 6.23 μIU/mL in the High-TSH group (**Table 2**). Additional analyses using all available measurements (n = 1,666) yielded similar results as shown in **Supplementary Figure 1**.

**Table 2:** Thyroid hormone and Tg levels among the low-, normal- and high-TSH groups.

TSH groups	TSH (μIU/mL; n=292)	FT4 (ng/dL; n=292)	FT3 (pg/mL; n=292)	Tg (ng/mL; n=292)
Reference range	0.61 - 4.23	0.97 - 1.69	2.30 - 4.00	3.71 - 35.12
Low-TSH (n=63)	0.01 [0.01-0.22] ^a^	1.66 [1.27-2.65] ^a^	3.65 [2.89-7.66] ^a^	42.68 [11.88-135.95] ^a^
Normal-TSH (n=191)	1.65 [1.08-2.48] ^b^	1.23 [1.13-1.35] ^b^	2.84 [2.61-3.07] ^b^	16.87 [5.45-39.25] ^b^
High-TSH (n=38)	6.23 [5.00-17.3] ^c^	1.07 [0.90-1.27] ^c^	2.57 [2.20-2.92] ^c^	12.23 [4.03-28.35] ^b^
*p* value	<0.01	<0.01	<0.01	<0.01

The values presented in **Table 2**, along with the bracketed values [ ], represent the median and interquartile range (IQR). Comparisons among the Low-, Normal-, and High-TSH groups were performed using the Kruskal–Wallis test. Because statistically significant differences were observed in **Table 2** (p < 0.01), post-hoc pairwise comparisons were subsequently performed using the Steel–Dwass method. The superscript letters (a, b, c) in **Table 2** indicate significant differences in post hoc pairwise comparisons; groups sharing the same letter are not significantly different. Values with different superscript letters indicate significant differences at p < 0.05. TSH, thyroid-stimulating hormone; FT4, free thyroxine; FT3, free triiodothyronine; Tg, thyroglobulin.

### 3.3 Distribution of serum Tg levels by TSH group

**Figure 2:**
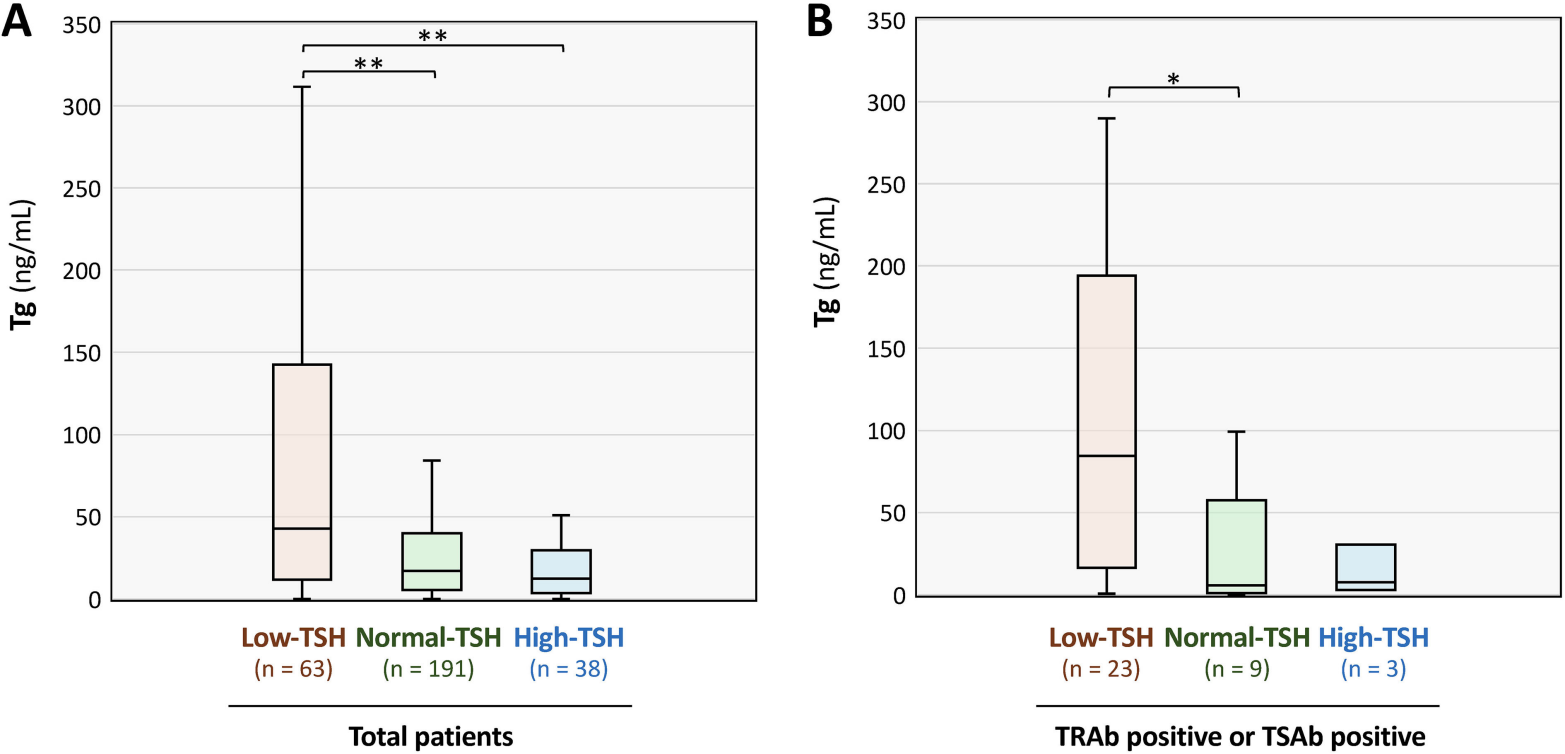
Distribution of serum thyroglobulin (Tg) levels among the high-, normal-, and low-thyroid-stimulating hormone (TSH) groups. **(A)** Serum Tg concentrations in all 292 patients, stratified according to TSH-based definitions described in **Figure 1**. **(B)** Serum Tg levels in patients positive for TSH-receptor antibody (TRAb) or thyroid-stimulating antibody (TSAb), including those tested for only one of the two autoantibodies. The High-, Normal-, and Low-TSH groups consisted of 3, 9, and 23 patients, respectively. In each panel, the top and bottom edges of the box indicate the 75th and 25th percentiles, respectively. The horizontal line within the box represents the median. The upper and lower whiskers represent the maximum and minimum values within the interquartile range. The Kruskal–Wallis test was applied to compare the groups, and as all comparisons showed statistically significant differences (*p* < 0.05), *post-hoc* analysis was subsequently performed using the Steel–Dwass test. **p < 0.01 and *p < 0.05 indicate statistically significant differences between groups.

Serum Tg levels were compared across the three TSH-defined groups using the Kruskal–Wallis test (**Figure 2**). *Post-hoc* analysis using the Steel–Dwass method revealed that Tg levels in the Low-TSH group were significantly higher than those in the Normal- and High-TSH groups(**Figure 2A**). Median serum Tg levels [interquartile range (IQR)] were 42.68 ng/mL (11.88–135.95) in the Low-TSH group, 16.87 ng/mL (5.45–39.25) in the Normal-TSH group, and 12.23 ng/mL (4.03–28.35) in the High-TSH group.

We then examined autoantibodies typically associated with Graves disease (TRAb and TSAb). Both TRAb and TSAb were significantly higher in the Low-TSH group than in the Normal-TSH group. Median TRAb levels were 6.5 IU/L in the Low-TSH group and 1.03 IU/L in the Normal-TSH group, whereas median TSAb levels were 606% and 105%, respectively (**Table 3**). These findings suggest that the primary pathology in the Low-TSH group is enriched for autoimmune hyperthyroidism. Based on these findings, we analyzed serum Tg levels in the subgroup of patients positive for either TRAb or TSAb, including those with only one measured antibody. Tg levels were higher in the Low-TSH [84.59 ng/mL (19.73–185.15)] than in the Normal-TSH [5.61 ng/mL; (1.98–16.08)] and High-TSH groups [7.49 ng/mL (5.20–18.90); **Figure 2B**].

**Table 3:** TRAB and TSAb levels among the low-, normal- and high-TSH groups.

TSH groups	TRAb (IU/L; n=93)		TSH groups	TSAb (%; n=55)
Reference range	< 2.0		Reference range	< 120
Low-TSH (n=34)	6.5 [1.48-12.4] ^a^		Low-TSH (n=26)	606 [128.75-2279] ^a^
Normal-TSH (n=50)	1.03 [0.8-1.41] ^b^		Normal-TSH (n=25)	105 [96-149] ^b^
High-TSH (n=9)	1.22 [0.97-1.48] ^ab^		High-TSH (n=4)	112.5 [103.75-122.25] ^ab^
*p* value	<0.01		*p* value	<0.01

The values presented in **Table 3**, along with the bracketed values [ ], represent the median and interquartile range (IQR). Comparisons among the Low-, Normal-, and High-TSH groups were performed using the Kruskal–Wallis test. Because statistically significant differences were observed in **Table 3** (p < 0.01), post-hoc pairwise comparisons were subsequently performed using the Steel–Dwass method. The superscript letters (a, b) in **Table 3** indicate significant differences in post hoc pairwise comparisons; groups sharing the same letter are not significantly different. Values with different superscript letters indicate significant differences at p < 0.05. TSH, thyroid-stimulating hormone; TRAb, TSH-receptor antibody; TSAb, thyroid-stimulating antibody.

We also examined the distribution of autoantibodies typically associated with Hashimoto thyroiditis (TPOAb and TgAb) across the three TSH-based groups (**Table 4**). No statistically significant differences were observed in TgAb levels among the groups, with median values of 15.5 IU/mL in the Low-TSH group, 11.2 IU/mL in the Normal-TSH group, and 13.5 IU/mL in the High-TSH group. In contrast, TPOAb levels showed a statistically significant difference among the three groups when analyzed using the Kruskal–Wallis test. However, *post-hoc* analysis using the Steel–Dwass method did not reveal any significant differences between specific group pairs. The median TPOAb levels were 48.1 IU/mL in the Low-TSH group, 3.4 IU/mL in the Normal-TSH group, and 5.4 IU/mL in the High-TSH group.

**Table 4:** TPO-Ab and Tg-Ab levels among the low-, normal- and high-TSH groups.

TSH groups	TPOAb (IU/mL; n=94)		TSH groups	TgAb (IU/mL; n=96)
Reference range	< 3.3		Reference range	< 19.3
Low-TSH (n=13)	48.1 [3.9-380.1] ^a^		Low-TSH (n=15)	15.5 [10-96.9] ^a^
Normal-TSH (n=58)	3.4 [2.43-41.15] ^ab^		Normal-TSH (n=59)	11.2 [10-263.3] ^a^
High-TSH (n=23)	5.4 [3.45-546.4] ^ab^		High-TSH (n=22)	13.5 [10-671.2] ^a^
*p* value	<0.05		*p* value	0.61

The values presented in **Table 4**, along with the bracketed values [ ], represent the median and interquartile range (IQR). Comparisons among the Low-, Normal-, and High-TSH groups were performed using the Kruskal–Wallis test. In **Table 4**, Kruskal–Wallis tests were performed for both TPOAb and TgAb. Although a significant difference was found for TPOAb (p < 0.05), post-hoc analysis using the Steel–Dwass test revealed no significant differences between any pair of groups. No significant difference was observed among the three groups for TgAb. The superscript letters (a, b) in **Table 4** indicate significant differences in post hoc pairwise comparisons; groups sharing the same letter are not significantly different. Values with different superscript letters indicate significant differences at p < 0.05. TSH, thyroid-stimulating hormone; TPOAb, Thyroid peroxidase antibody; TgAb, thyroglobulin antibody.

### 3.4 Correlations of serum Tg and clinical parameters stratified by TSH levels

**Figure 3:**
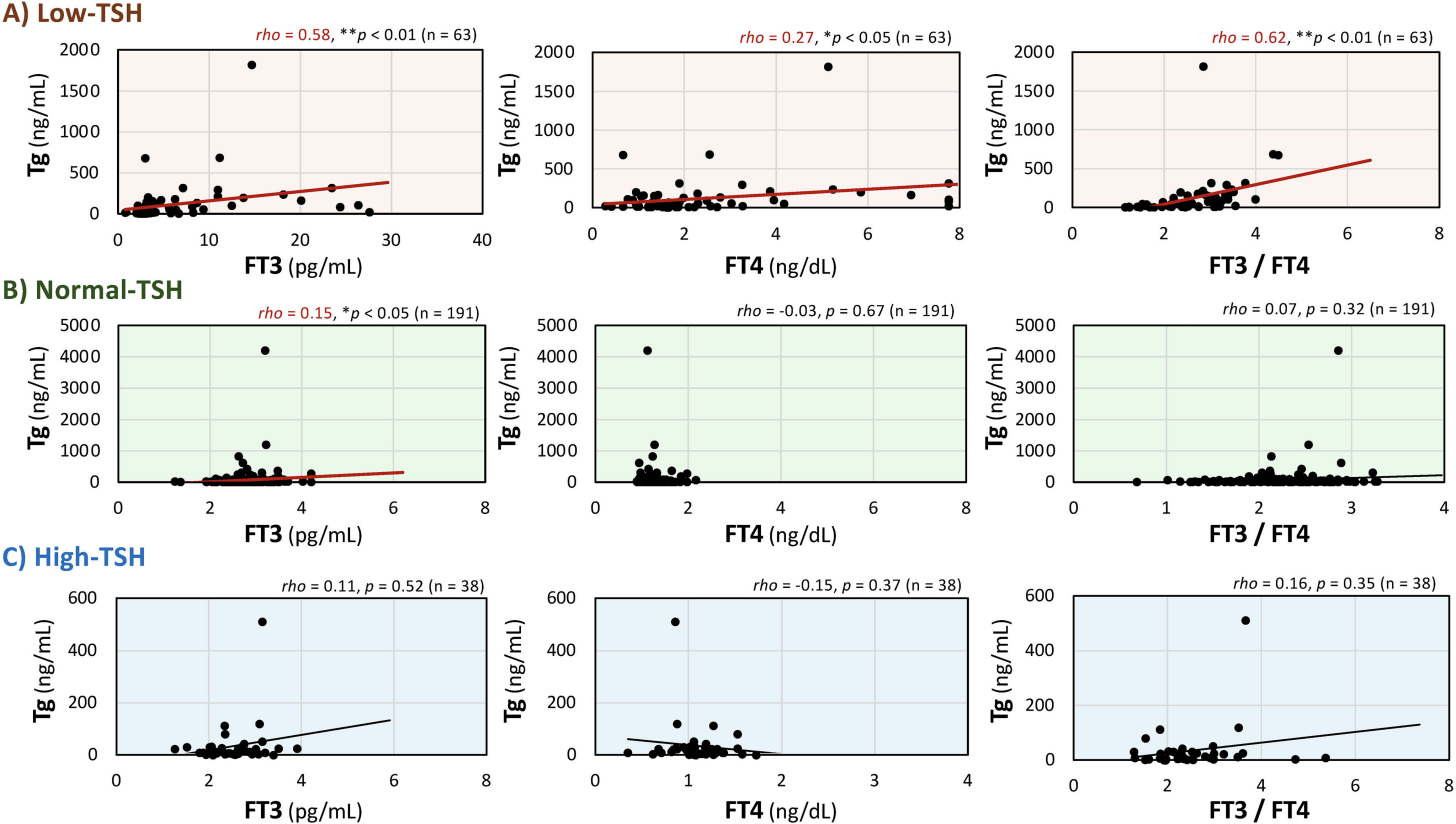
Correlations between serum thyroglobulin (Tg) levels and free triiodothyronine (FT3), free thyroxine (FT4), and the FT3/FT4 ratio in the low-, normal-, and high-thyroid-stimulating hormone (TSH) groups. Scatter plots illustrate the relationships between serum Tg and FT3, FT4, and the FT3/FT4 ratio in the Low-TSH group **(A)**, Normal-TSH group **(B)**, and High-TSH group **(C)**. Since the data were not normally distributed, correlations were evaluated using Spearman’s rank correlation coefficient. Red regression lines indicate statistically significant correlations. **p < 0.01 and *p < 0.05 indicate statistically significant differences between groups.

The relationships between serum Tg levels and FT3, FT4, and the FT3/FT4 ratio in each group are shown in **Figure 3**. In the Low-TSH group, Tg levels were positively and significantly correlated with FT3, FT4, and the FT3/FT4 ratio. The positive correlation was observed with the FT3/FT4 ratio, FT3 and FT4 (Tg × FT3/FT4 ratio: *rho* = 0.62, *p* < 0.01; Tg × FT3: *rho* = 0.58, *p* < 0.01; Tg × FT4: *rho* = 0.27, *p* < 0.05; **Figure 3A**). In contrast, in the Normal-TSH group, Tg levels showed statistically significant positive correlation with FT3 (*rho* = 0.15, *p* < 0.05), whereas no significant correlations were observed with FT4 (*rho* = –0.03, *p* = 0.67) or the FT3/FT4 ratio (*rho* = 0.07, *p* = 0.32; **Figure 3B**). Similarly, in the High-TSH group, no significant correlations were observed between Tg and FT3 (*rho* = 0.11, *p* = 0.52), FT4 (*rho* = –0.15, *p* = 0.37), or the FT3/FT4 ratio (*rho* = 0.16, *p* = 0.35; **Figure 3C**).

### 3.5 Sensitivity analysis excluding extreme Tg values

To assess the potential influence of outliers, additional analyses were performed using data restricted to serum Tg levels < 500 ng/mL. Under this condition, the relationships between Tg and FT3, FT4, and the FT3/FT4 ratio across the Low-, Normal-, and High-TSH groups were similar to those observed in the overall dataset(**Supplementary Figure 2**). No meaningful differences were observed in the direction or statistical significance of these associations.

### 3.6 Distribution of serum Tg levels in subgroups defined by the TSH-receptor antibodies

**Figure 4:**
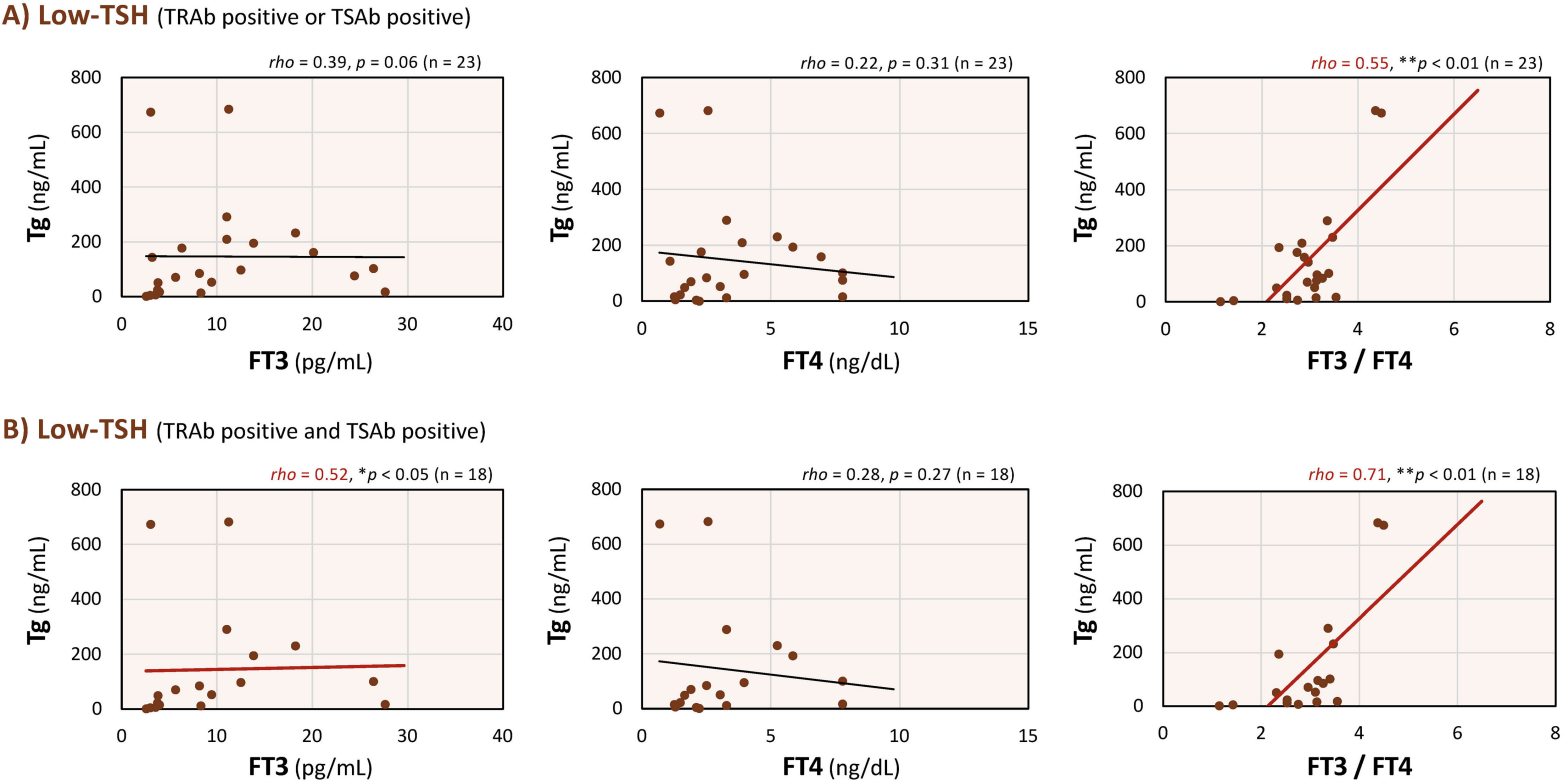
Correlations between serum thyroglobulin (Tg) levels and free triiodothyronine (FT3), free thyroxine (FT4), and the FT3/FT4 ratio in low-thyroid-stimulating hormone (TSH) patients with positive TSH-receptor antibody (TRAb) or thyroid-stimulating antibody (TSAb). Associations between serum Tg and each thyroid hormone parameter in patients positive for either TRAb or TSAb **(A)**, and for both TRAb and TSAb **(B)**. As the data did not follow a normal distribution, Spearman’s rank-order correlation was used to assess the relationships between variables. Red regression lines indicate statistically significant correlations within each panel. **p < 0.01 and *p < 0.05 indicate statistically significant differences between groups.

Finally, correlations were examined in patients positive for TRAb or TSAb. As shown in **Figure 4**, in both the single-positive (TRAb or TSAb) and double-positive (TRAb and TSAb) subgroups, serum Tg levels showed significant positive correlations with the FT3/FT4 ratio. In the double-positive subgroup, Tg was also significantly correlated with FT3 (*rho* = 0.52, *p* < 0.05). The correlation coefficient between Tg and the FT3/FT4 ratio was 0.71 (*p* < 0.01) in the double-positive subgroup and 0.55 (*p* < 0.01) in the single-positive subgroup (**Figure 4**). Among patients positive for either TRAb or TSAb, no significant correlations were observed between Tg and FT3 (*rho* = 0.39, *p* = 0.06) or FT4 (*rho* = 0.22, *p* = 0.31; **Figure 4A**). In the double-positive subgroup, Tg levels were not correlated with FT4 (*rho* = 0.28, *p* = 0.27), although correlations with other parameters were observed (**Figure 4B**).

### 3.7 Multivariable regression analysis of the association between Tg and the FT3/FT4 ratio

To further evaluate this association, multivariable regression analyses were performed. Across all models, log-transformed Tg remained consistently and independently associated with the log-transformed FT3/FT4 ratio (Crude model: *β* = 0.053, *p* < 0.01; Model I: *β* = 0.056, *p* < 0.01; Model II: *β* = 0.056, *p* < 0.01; Model III: *β* = 0.057, *p* < 0.01; **Table 5**). Model I was adjusted for age, sex, and FT4, Model II additionally included TRAb status, and Model III included TSAb status. In all models, the association between Tg and the FT3/FT4 ratio remained significant after adjustment for these covariates. These findings indicate that the observed association between Tg and the FT3/FT4 ratio is independent of the covariates included in the models.

**Table 5:** Multivariable regression analysis of log-transformed Tg and FT3/FT4 ratio.

	Crude model		Model I		Model II		Model III	
	*β-*coefficients (95% CI)	*p* value	*β-*coefficients (95% CI)	*p* value	*β-*coefficients (95% CI)	*p* value	*β*-coefficients (95% CI)	*p* value
Log-transformed Tg	0.053 (0.039 − 0.068)	< 0.01	0.056 (0.041 − 0.070)	< 0.01	0.056 (0.041 – 0.071)	< 0.01	0.057 (0.042 - 0.071)	< 0.01

In **Table 5**, Crude model: unadjusted model including log-transformed Tg. Model I: adjusted for age, sex, and FT4. Model II: Model I additionally adjusted for TRAb status. Model III: Model I additionally adjusted for TSAb status. TSH, thyroid-stimulating hormone; FT4, free thyroxine; Tg, thyroglobulin; TRAb, TSH-receptor antibody; TSAb, thyroid-stimulating antibody.p<0.05 indicates statistically significant.

### 3.8 Correlations between serum Tg levels and SPINA-GT and SPINA-GD in the Low-TSH group

**Figure 5:**
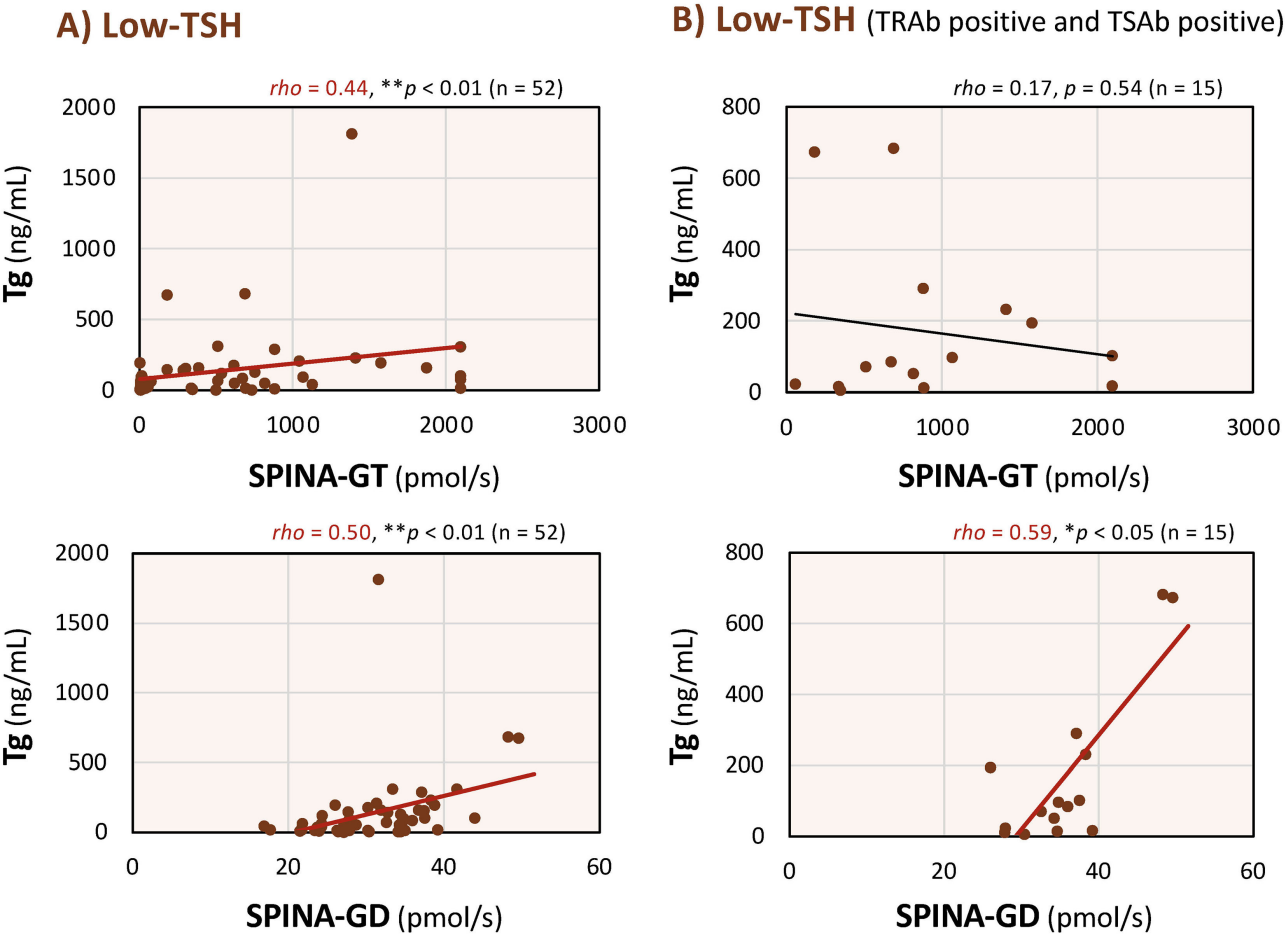
Correlations between serum thyroglobulin (Tg) levels and thyroid’s secretory capacity (SPINA-GT) and the sum activity of peripheral deiodinases (SPINA-GD) in the Low-thyroid-stimulating hormone (TSH) group, including all patients regardless of antibody status and those positive for TSH-receptor antibodies. Associations between serum Tg levels and SPINA-GT and SPINA-GD in the Low-TSH group, analyzed in all patients **(A)** and in those positive for TSH-receptor antibody (TRAb) and thyroid-stimulating antibody (TSAb) **(B)**. As the data did not follow a normal distribution, Spearman’s rank-order correlation was used to assess the relationships between variables. Red regression lines indicate statistically significant correlations within each panel. **p < 0.01 and *p < 0.05 indicate statistically significant differences between groups.

To further explore the association between Tg and thyroid hormone dynamics, and based on the positive correlations between Tg and each parameter observed in the Low-TSH group in **Figure 3**, correlations between serum Tg levels and SPINA-GT and SPINA-GD, the model-based indices of thyroid function, reflecting secretory capacity and deiodinase activity, were evaluated in the Low-TSH group (**Figure 5**). As shown in **Figure 5A**, under the condition of Low-TSH, both SPINA-GT and SPINA-GD were positively correlated with serum Tg levels (Tg × SPINA-GT: *rho* = 0.44, *p* < 0.01; Tg × SPINA-GD: *rho* = 0.50, *p* < 0.01). In contrast, in patients positive for both TRAb and TSAb, only SPINA-GD showed a significant positive correlation with Tg (Tg × SPINA-GT: *rho* = 0.17, *p* = 0.54; Tg × SPINA-GD: *rho* = 0.59, *p* < 0.05; **Figure 5B**). These results suggest that in a low-TSH state, Tg may reflect the dynamics of thyroid hormones as captured by SPINA parameters, particularly SPINA-GD, and this trend is more pronounced in TRAb and TSAb-positive individuals.

### 3.9 Disease-specific correlations between serum Tg levels and clinical parameters in the Low-TSH group of autoimmune thyroid diseases

**Figure 6:**
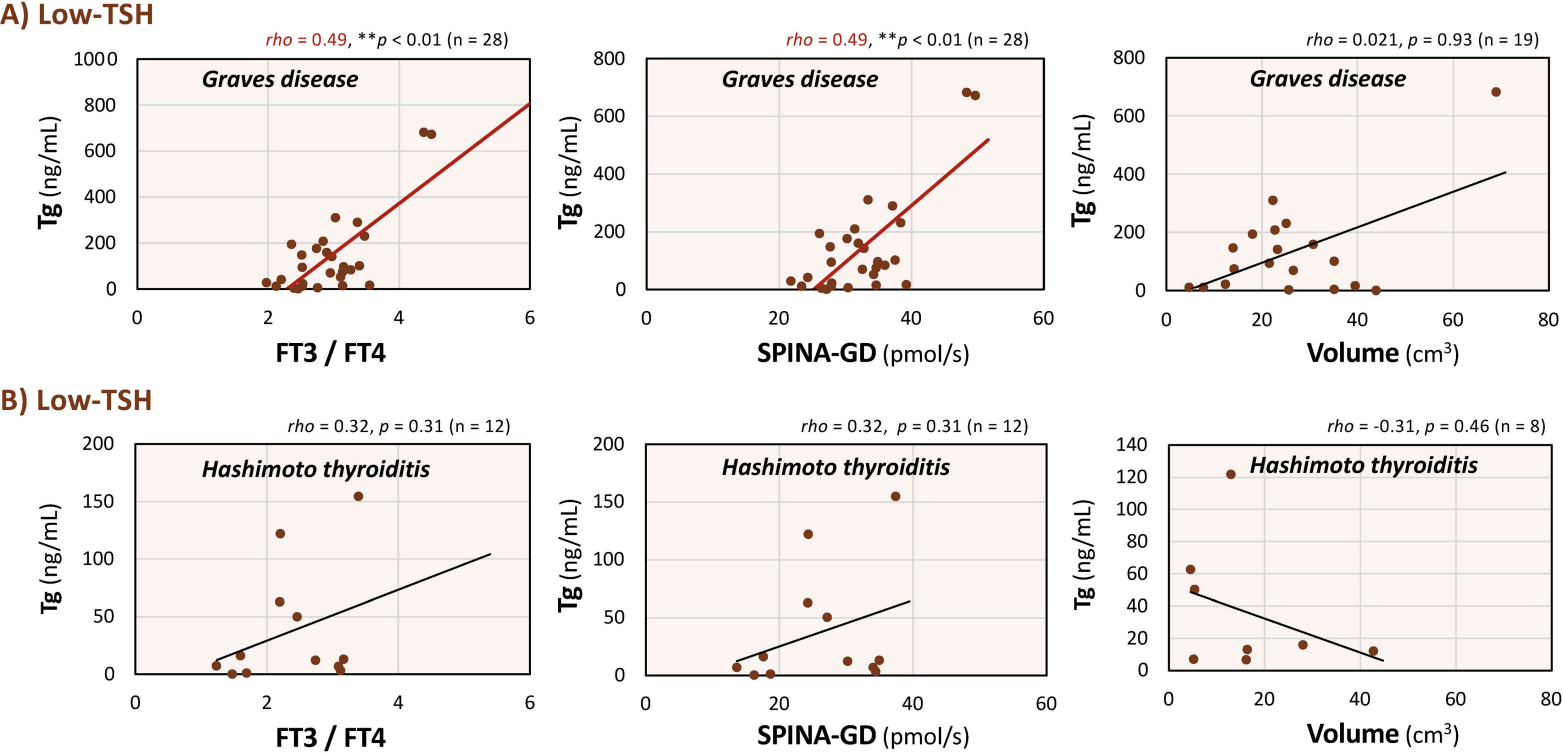
Correlations between serum thyroglobulin (Tg) levels and the free triiodothyronine (FT3)/free thyroxine (FT4) ratio, the sum activity of peripheral deiodinases (SPINA-GD), and thyroid volume in the low-thyroid-stimulating hormone (TSH) group according to autoimmune thyroid diseases. Relationships between serum Tg levels and each parameter within the Low-TSH group in Graves disease **(A)** and Hashimoto thyroiditis **(B)**. As the data did not follow a normal distribution, Spearman’s rank-order correlation was used to assess the relationships between variables. Red regression lines indicate statistically significant correlations within each panel. **p < 0.01 indicate statistically significant differences between groups.

Given that detailed clinical information and ultrasonographic data were available, additional analyses were performed to evaluate disease-specific correlations between serum Tg levels and clinical parameters in the Low-TSH group of autoimmune thyroid diseases(**Figure 6**). In patients with Graves disease, Tg was significantly correlated with the FT3/FT4 ratio and SPINA-GD (Tg × FT3/FT4: *rho* = 0.49, *p* < 0.01; Tg × SPINA-GD: *rho* = 0.49, *p* < 0.01), whereas no significant correlation was observed between Tg and thyroid volume (Tg × Volume: *rho* = 0.021, *p* = 0.93; **Figure 6A**). In contrast, no significant correlations were observed between serum Tg levels and any of the parameters in patients with Hashimoto thyroiditis (Tg × FT3/FT4: *rho* = 0.32, *p* = 0.31; Tg × SPINA-GD: *rho* = 0.32, *p* = 0.31; Tg × Volume: *rho* = -0.31, *p* = 0.46; **Figure 6B**). These results support the significance of the association between serum Tg levels and the FT3/FT4 ratio and the disease-specific characteristics in the low-TSH group.

## 4 Discussion

In the present study, we investigated the interrelationships between serum Tg levels and thyroid function parameters across different TSH categories. The results revealed that in the low-TSH group, Tg levels showed a positive correlation with the FT3/FT4 ratio, FT3, and FT4, with the strongest association observed for the FT3/FT4 ratio. The correlation with the FT3/FT4 ratio was more evident in patients who tested positive for both TRAb and TSAb. These findings suggest that serum Tg levels are associated with biochemical features of thyroid hormone metabolism, including indices related to T3 predominance. Given that this association was observed in patients positive for thyroid-stimulating antibodies, Tg may be associated with biochemical features consistent with increased T3 production in Graves disease.

Of interest, the positive correlations observed between serum Tg and thyroid hormone parameters in the low-TSH group are consistent with the established physiological role of Tg in thyroid hormone synthesis and secretion. While serum Tg levels can rise during thyroid tissue destruction (22), elevated Tg levels have also been reported in non-destructive hyperthyroid conditions such as Graves disease (23). Although the underlying mechanism remains uncertain, Tg has been proposed to participate in an intrinsic regulatory role during thyroid hormone biosynthesis (24, 25), possibly increasing in response to excessive hormone production as part of a compensatory or autoregulatory process.

Because clinical and biochemical findings may overlap, distinguishing Graves disease from destructive thyroiditis (e.g., painless or subacute thyroiditis) can be challenging when based on laboratory data alone. Imaging modalities such as thyroid ultrasonography or scintigraphy are often required to confirm the diagnosis (26, 27). However, these techniques may not always be readily available in routine clinical practice. Therefore, differentiating various causes of thyrotoxicosis based solely on laboratory data remains a long-standing diagnostic challenge. Given that FT3 levels tend to be disproportionately higher than FT4 levels in Graves disease compared with other etiologies of thyrotoxicosis, the FT3/FT4 ratio has been investigated as a useful diagnostic index (28, 29). Our findings suggest that Tg levels, particularly in patients with suppressed TSH, may further improve diagnostic discrimination among different thyrotoxic states. Under physiologic conditions, Tg secretion is biphasically regulated by TSH, diminishing once stimulation exceeds a specific threshold. In contrast, Tg secretion increases in a dose-dependent manner in response to TSAb stimulation (30). These contrasting regulatory mechanisms underscore the potential pathophysiological differences and highlight the diagnostic relevance of Tg measurement in evaluating thyrotoxic states.

In this regard, Graves disease has been associated with increased activities of iodothyronine deiodinases, particularly type 1 (D1), in thyroid follicular cells (31). D1 and D2 catalyze the conversion of T4 to T3, although D1 predominantly contributes to circulating T3 production, whereas D2 primarily regulates intracellular T3 concentrations (32). In Graves disease, stimulation of the TSH receptor by autoantibodies enhances intracellular cyclic AMP signaling, which in turn upregulates the expression of these deiodinases, especially D1 (33). This mechanism contributes to the disproportionately elevated FT3 relative to FT4 observed in autoimmune thyrotoxicosis. Accordingly, the FT3/FT4 ratio has been reported to correlate with intrathyroidal D1 activity in Graves disease (34, 35), consistent with the enzymatic basis of T4-to-T3 conversion.

Notably, in the present study, a positive correlation between the FT3/FT4 ratio and serum Tg levels was observed in patients positive for both TRAb and TSAb. In particular, the antibody positivity is consistent with an autoimmune hyperthyroid state, and under conditions of suppressed TSH, serum Tg levels may be associated with biochemical features related to T4-to-T3 conversion. A recent investigation reported lower Tg levels in Graves disease than in other thyroid disorders; however, that study described this as a limitation related to the presence of TgAb. (36). In contrast, our study employed a Tg measurement method specifically designed to minimize TgAb interference, thereby enhancing the reliability of Tg quantification (18). Nevertheless, because deiodinase activity was not directly measured, further studies are required to clarify the mechanistic relationship between serum Tg and iodothyronine deiodinase activities.

With advancements in immunoassay technology, the precision of Tg measurement has markedly improved (37, 38), warranting renewed consideration of its role in the clinical assessment of thyrotoxicosis. Beyond its established role as a structural or tumor marker, serum Tg may warrant re-evaluation as a potential dynamic indicator of thyroid hormone metabolism. However, the current study has several limitations. First, not all patients underwent antibody testing or imaging studies, and therefore the underlying etiology of thyroid dysfunction could not be definitively established in all cases. This is an inherent limitation of the retrospective study design. Second, detailed data on potential confounding factors, including thyroid volume, iodine intake, and medication exposure, were not fully available. Third, although serum Tg was measured using an assay designed to minimize interference from anti-thyroglobulin antibodies (TgAb), such interference cannot be completely excluded, and Tg values in TgAb-positive patients should be interpreted with caution. Fourth, analyses performed after excluding extreme Tg values (Tg ≥ 500 ng/mL) to assess the influence of outliers, as well as subgroup analyses restricted to antibody-positive patients, were limited by relatively small sample sizes, which may have reduced statistical power. Finally, although values below or above the detection limits were substituted with the corresponding limits and the proportion of such measurements was low, the exact values could not be determined, which may introduce a degree of measurement uncertainty.

In conclusion, this study demonstrated a significant correlation between serum Tg levels and thyroid function in the low-TSH group, particularly with the FT3/FT4 ratio among patients positive for both TRAb and TSAb. These findings suggest that serum Tg may reflect biochemical features consistent with T3 predominance in autoimmune hyperthyroidism, although confirmation in future pathophysiological studies is required. Furthermore, Tg measurement may provide additional insights for clinical decision-making, optimizing therapeutic strategies in patients with hyperthyroidism.

## Data Availability

The raw data supporting the conclusions of this article will be made available by the authors, without undue reservation.

## Supplementary material

Supplementary Figure 1 Distribution of serum thyroid-stimulating hormone (TSH) and free thyroxine (FT4) levels among the study population. Patients were categorized into three groups based on serum TSH concentrations: High-TSH (> 4.23 μIU/mL), Normal-TSH (0.61–4.23 μIU/mL), and Low-TSH (< 0.61 μIU/mL). Distribution of serum TSH and FT4 levels across all 1,666 measurements obtained from the same 292 patients. The High-, Normal-, and Low-TSH groups represented 16% (n = 262), 61% (n = 1,018), and 23% (n = 386) of total measurements, respectively.

Supplementary Figure 2 Under the condition of serum thyroglobulin (Tg) levels < 500 ng/mL, relationships between Tg and free triiodothyronine (FT3), free thyroxine (FT4), and the FT3/FT4 ratio are shown for the low-, normal-, and high-thyroid-stimulating hormone (TSH) groups. Scatter plots depict the associations between Tg and each parameter across the three TSH groups: Low-TSH group **(A)**, Normal-TSH group **(B)**, and High-TSH group **(C)**. As the data were not normally distributed, Spearman’s rank correlation coefficient was used to assess these relationships. Regression lines are shown in red where statistically significant correlations were identified. ***p* < 0.01 and **p* < 0.05 denote statistical significance.
