# Supplementary figures and images for: Behavior of serum thyroglobulin in relation to thyroid function under low-thyrotropin conditions in general practice
Source: Front Endocrinol (Lausanne). 2026 May 15;17:1756863. doi: 10.3389/fendo.2026.1756863 (PMC13218918; doi:10.3389/fendo.2026.1756863)

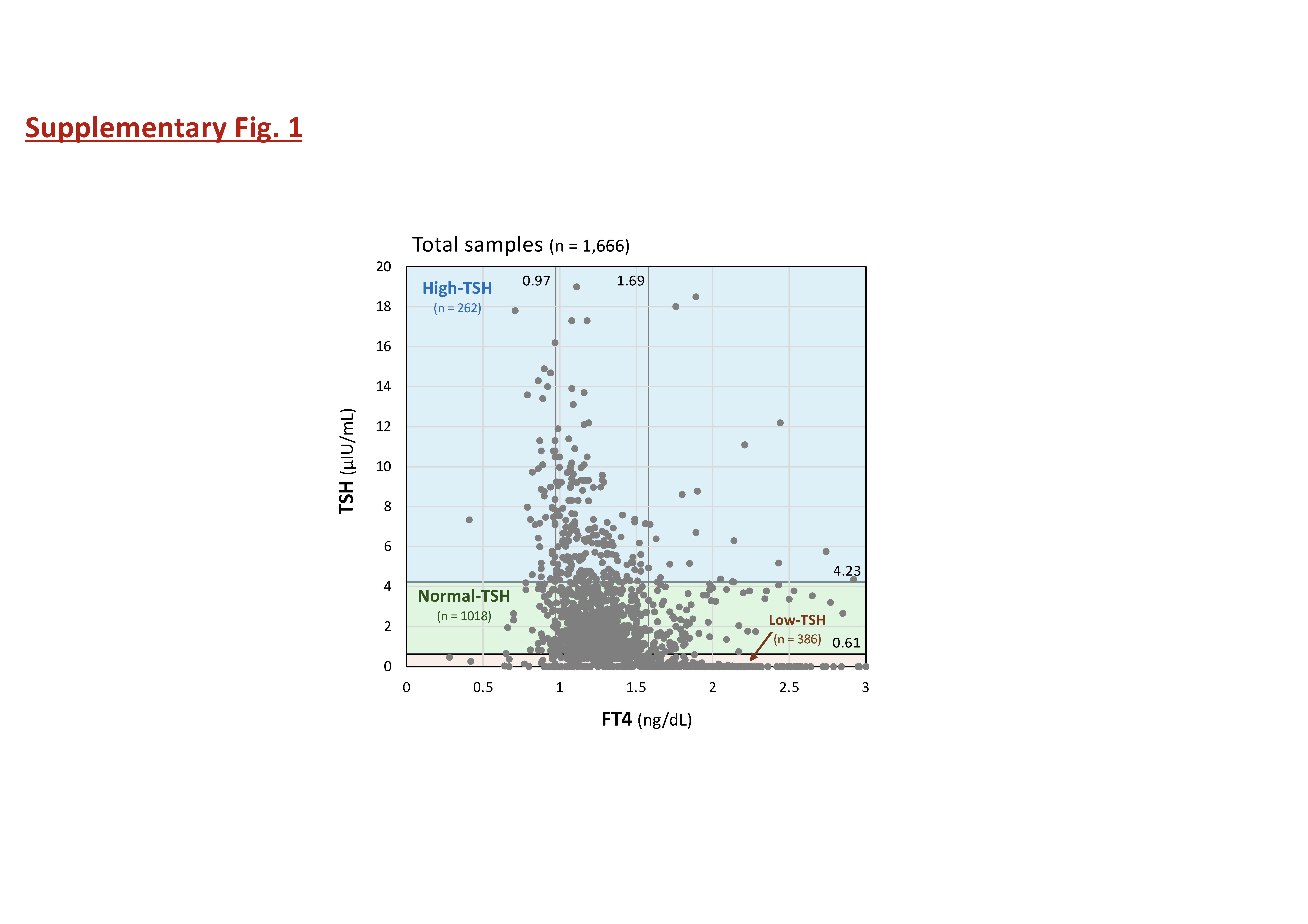

Supplement: Supplementary Figure 1 — Distribution of serum thyroid-stimulating hormone (TSH) and free thyroxine (FT4) levels among the study population. Patients were categorized into three groups based on serum TSH concentrations: High-TSH (> 4.23 μIU/mL), Normal-TSH (0.61–4.23 μIU/mL), and Low-TSH (< 0.61 μIU/mL). Distribution of serum TSH and FT4 levels across all 1,666 measurements obtained from the same 292 patients. The High-, Normal-, and Low-TSH groups represented 16% (n = 262), 61% (n = 1,018), and 23% (n = 386) of total measurements, respectively. [file Image1.jpeg]

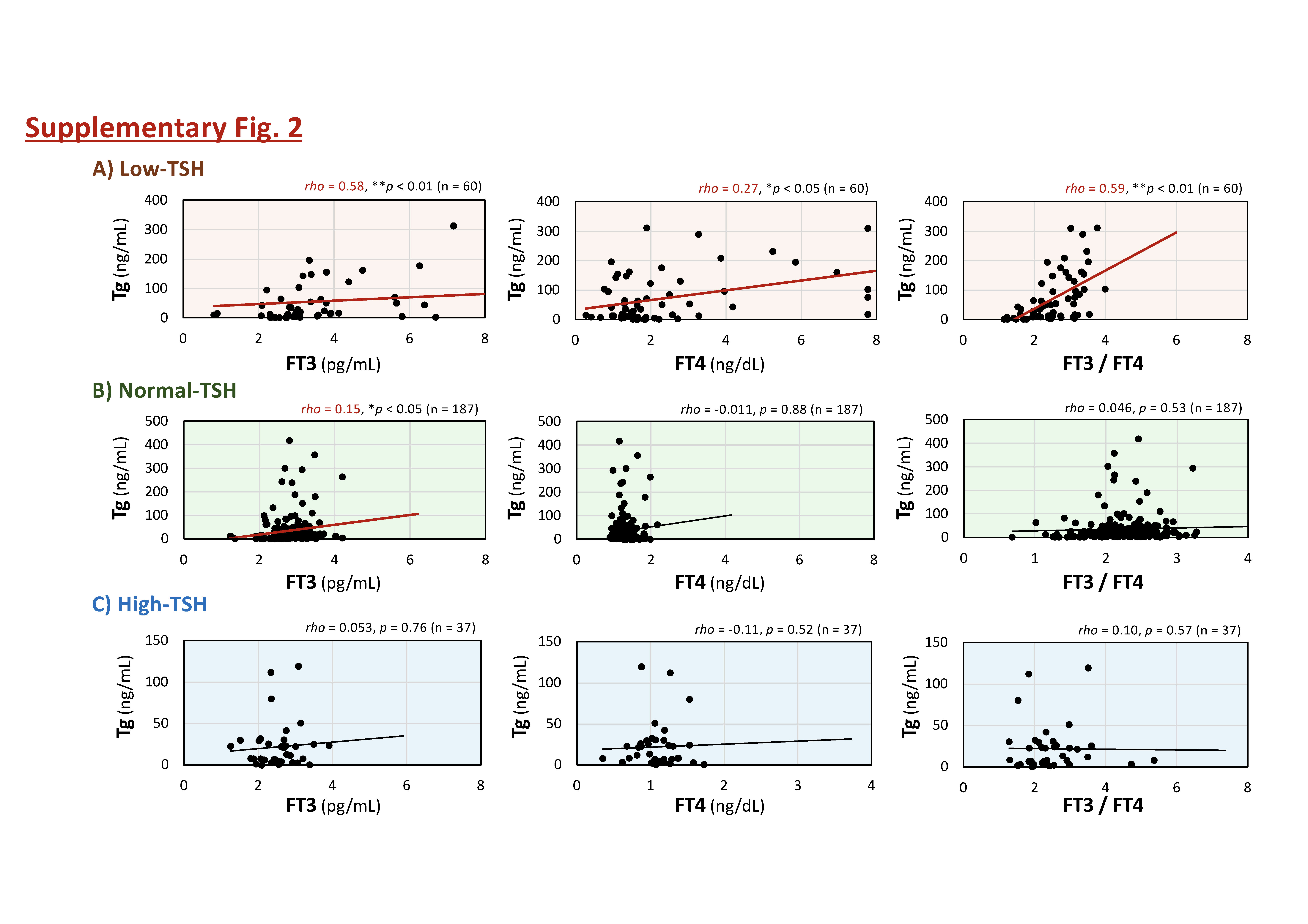

Supplement: Supplementary Figure 2 — Under the condition of serum thyroglobulin (Tg) levels < 500 ng/mL, relationships between Tg and free triiodothyronine (FT3), free thyroxine (FT4), and the FT3/FT4 ratio are shown for the low-, normal-, and high-thyroid-stimulating hormone (TSH) groups. Scatter plots depict the associations between Tg and each parameter across the three TSH groups: Low-TSH group (A), Normal-TSH group (B), and High-TSH group (C). As the data were not normally distributed, Spearman’s rank correlation coefficient was used to assess these relationships. Regression lines are shown in red where statistically significant correlations were identified. **p < 0.01 and *p < 0.05 denote statistical significance. [file Image2.jpeg]
